# Lengths of inpatient stay and sick leave of patients with mental diseases: disorder-specific effects of flexible and integrated treatment programs in Germany

**DOI:** 10.1038/s41398-022-02131-5

**Published:** 2022-09-07

**Authors:** Fabian Baum, Jochen Schmitt, Martin Seifert, Roman Kliemt, Denise Kubat, Stefanie March, Dennis Häckl, Andrea Pfennig, Enno Swart, Anne Neumann

**Affiliations:** 1 Center of Evidence-based Health Care, Medizinische Fakultät Carl Gustav Carus, Technische Universität Dresden, Dresden, Germany; 2 WIG2 Scientific Institute for Health Economics and Health System Research Leipzig, Leipzig, Germany; 3 Institute of Social Medicine and and Health Systems Research, Medical Faculty, Otto-von-Guericke- University Magdeburg, Magdeburg, Germany; 4 Hochschule Magdeburg-Stendal, Department of Social Work, Health and Media, Magdeburg, Germany; 5 Health Economics and Management, Faculty of Economics and Management Science, Leipzig University, Leipzig, Germany; 6 Department of Psychiatry and Psychotherapy, Carl Gustav Carus University Hospital, Technische Universität Dresden, Dresden, Germany

**Keywords:** Psychiatric disorders, Depression, Schizophrenia

## Abstract

Mental disorders pose a worldwide growing public health burden. One of the major challenges for healthcare systems remains to respond to the need of patients with mental disorders for continuous and flexible treatment. The EVA64 study evaluates novel programs of flexible and integrative treatment (FIT) in hospitals. This manuscript presents results from the evaluation of FIT hospitals in comparison to hospitals from regular routine care. In addition to data from adult patients, we also present data from affiliated child and adolescent psychiatric wards employing FIT programs. Using comprehensive claims data, primary outcomes are the utilization of inpatient care and sick leave for a priori defined clusters of mental disorders. We stratify between patients already under treatment (ongoing treatment) and patients with incident treatment cases (initial treatment) at the point of inclusion in the study. In the initial treatment group, we found a significant reduction in the length of inpatient stay of 4.1 days in FIT hospitals compared to routine care. While patients with mood affective disorders (−1.8 days) and patients with neurotic, stress-related, and somatoform disorders (−3.6 days) showed an even stronger effect of the reduction of inpatient lengths of stay, the effect was significantly weaker in patients with mental and behavioral disorders due to use of alcohol (+3.3 days). Regarding the duration of sick leave, we found no significant treatment effect of FIT programs compared to routine care. In the ongoing treatment group of adult patients, we found a significantly lower utilization of inpatient treatment by 1.3 days as well as a shorter duration of sick leave by 4.3 days in FIT hospitals compared to routine care. In the cohort of children and adolescent patients, we also did not observe a significant treatment effect in either the initial treatment group or the ongoing treatment group. Registration: this study was registered in the database “Health Services Research Germany” (trial number: VVfD_EVA64_15_003713).

## Introduction

Mental disorders pose a worldwide growing public health burden in terms of prevalence, cost, and morbidity [1]. However, there is still a gap between the actual need for treatment and its provision in many countries of the world, including Germany [2]. One of the major challenges for health care systems remains to adequately respond to the need of patients with mental disorders for adaptive patient-centered and continuous treatment [3, 4]. Standard care for patients with mental disorders in Germany, though, is currently still characterized by a strong focus on inpatient treatment [5, 6]. Furthermore, the German healthcare system suffers from insufficient interfaces between different sectors of health care, particularly in the field of psychiatric care [7, 8]. This goes together with a strong fragmentation of the financing system in German psychiatric health care even within the hospital sector: daily fee based budgets for inpatient and daycare services are strictly separated from the lump sum budget of the psychiatric outpatient departments (PIA). Furthermore, beyond hospital care, psychiatric outpatient services are financed by a variety of funds with different legal foundations and with different organizational and administrative structures. This financial separation constitutes an obstacle for efforts towards an efficient trans-sectoral treatment [9] resulting in misguided incentives, such as maximizing inpatient occupancies by admitting as many patients as possible with the highest possible retention time [10]. This notion is backed by data showing that Germany ranks number one with regard to average length of hospital stay for patients with mental disorders when compared to equally effective health care systems, such as Sweden, Norway, Denmark, or Netherlands [11]. Prioritization of inpatient capacity binds resources that otherwise would be available for PIA or outpatient treatment. Even more, it may lead to an inadequate follow-up treatment continuation by hindering integration of inpatient and outpatient treatment, psychotherapy, and psychosocial services and might even obstruct joint care approaches involving multiple medical specialists [8]. Lastly, long inpatient treatments might harm patients as they withdraw them from their normal living and social environment.

In response, there have been a number of initiatives promoting new financing budgets (global treatment budgets) aiming to promote patient-centered, cross-sectoral health care for mentally ill patients [7, 12–14]. All these novel projects share the common goal of providing a continuous, flexible, and integrated treatment to patients. The most recent legislative approach to flexible and integrated treatment (FIT) enabled statutory health insurance (SHI) funds, together with a total of 22 model hospitals (FIT hospitals) across Germany, to establish individual contracts of health care monetization. The contracts represent a hybrid installment of both capitation budget [12, 15–17] and block contract [18–20]. According to these contracts, each hospital has an overall fixed annual budget for all patients based on the number of patients treated in the previous year, including inpatient care, day care, and outpatient care. This budget covers all treatment expenses independent from the hospital setting, thus leaving room for the provider to apply an individual treatment strategy. These global treatment budgets are commonly thought to allow for a more flexible and integrated treatment, enabling innovative integrated treatment options provided by multi-professional teams. Such forms of treatment may include e.g., Assertive Community Treatment, Home-Treatment [21, 22], Crisis Resolution Teams [23], or a stronger focus on day-care treatment focussing on more need-adapted, cross-sectoral service [24]. FIT hospitals are enabled to configure models of care that suit the regional peculiarities and meet the needs of community members [25] and, therefore, tend to differ tremendously in terms of starting conditions as well as treatment and process structures (for more information please see e.g., [21, 24, 26–29]. Additionally, FIT programs may also vary on the degree of FIT implementation [24] as well as between adult psychiatry and children and adolescent psychiatry. Even so, adaptable cross-sectoral treatment options and flexible use of personnel are common to all FIT hospitals investigated here [24]. Therefore, all FIT programs are expected to reduce inpatient hospitalization of patients whenever possible and to strengthen non-inpatient treatment options, such as outpatient treatment in the hospital or home treatment, apart from their individual conditions. Further, these common implementation factors should also affect other critical factors in mental health care, such as improving continuity of treatment and reducing inpatient re-admission.

The overall evaluation study of FIT programs covering 18 FIT hospitals throughout Germany (EVA64) provides a standardized evaluation protocol for all model projects on a common scientific basis. As FIT programs represent complex interventions, the evaluation covers a set of eleven outcomes, such as duration of inpatient care, duration of sick leave, intensity of outpatient care, cross-sectoral treatment continuation, or inpatient re-admission rates [30]. In a first meta-analysis comprising 13 model hospitals it has been shown that the new financing models result in a shorter duration of inpatient treatment and a trend towards a reduced duration of sick leave days [31, 32]. However, this effect was only present for a sup-group of patients mostly representing incident treatment cases (hospital-new patients). For patients, that have been in treatment for or a longer time (hospital-known patients), no effect was found. While the reduction of inpatient treatment is one of the major goals of FIT programs, it is not a marker of more flexible treatment per se. Nonetheless, another analysis comprising 12 FIT hospitals and its controls also found that psychiatric care in hospital-new patients is seemingly shifted towards the outpatient and daycare sector [31, 33].

No attention has been payed so far regarding differential effects within different clusters of psychiatric disorders. Furthermore, no data from affiliated children and adolescent psychiatric clinics being part of FIT programs have been published before. This manuscript presents results of the EVA64 evaluation study focusing on data from the first evaluation year of all 18 FIT hospitals and its respective control hospitals. The goal is, first, to give a general estimate of the utilization of inpatient care and sick leave for a priori defined clusters of mental disorders and comparing them between patients in FIT hospitals and patients in hospitals employing routine care. For both outcomes, we hypothesized a relative reduction in patients treated in FIT hospitals when compared to the patients treated in routine care. Second, we were interested in differential variations of previously reported effects of global treatment budgets on these outcomes within various diagnostic clusters. While this is an exploratory research question, we can pose some assumptions about the nature of the intended effects. Differential effects within different categories of mental disorders are of special importance as diagnostic clusters differ with respect to inpatient treatment and in the inpatient length of stay [34] in the first place. Treatment of certain mental disorders like schizophrenia (especially in acute phases of positive symptom expression), or alcohol addiction (specifically in clinical guided withdrawal phases) might be specifically reliant on inpatient treatment leaving less room for alternative treatment options. On the other hand, diseases like depression or anxiety disorders might be more prone to avoiding hospitalization. Mental comorbidity additionally increases inpatient need of treatment [35, 36] as well as sick leave [37].

## Materials and methods

### Study design and population

The EVA64 study is a controlled cohort study. It employs a pre-post control group design based on claims data from SHI of Psychiatric wards, PIAs, and, if present, departments of children and adolescent psychiatry (CAP) from each of the evaluated 18 FIT hospitals and its matched control hospitals [30]. It utilizes anonymized patient data from German SHI funds [38] covering a total time span of 6 years with a 2-year pre-period and a 4-year post-period of adult and CAP patients with mental diseases. In this analysis, we used patient individual data covering a total time span of 2 years with 1 year prior (pre) and 1 year subsequent (post) to inclusion into the study. Note that an individual’s inclusion into the study is referenced by an index case indicating his or her first treatment in the index hospital after onset of the FIT program. Thus, the patient individual pre-time denotes an intervention-free time span with respect to the patient´s index hospital. FIT programs started between January 2013 and January 2017 in all analyzed hospitals. All 18 Fit hospitals and its consecutive control hospitals included a general psychiatric ward for adult patients. Additionally, five of the Fit hospitals (and hence, also its consecutive control hospitals) also included a ward for child and adolescent psychiatry. All patients insured by any of the participating SHI funds and treated in one of the FIT hospitals (IG, intervention group) are compared to control patients from hospitals of routine care (CG, control group) with respect to changes from the pre to the post time period. For each individual hospital, we defined two sub-groups based on pre-intervention health care utilization in that specific hospital. The initial treatment group comprised only patients that had no contact to the psychiatric ward or PIA in the corresponding FIT or control hospital in the 2 years prior to being included. The ongoing treatment group comprised patients that had to have at least one such contact during those 2 years. Note that patients in the initial treatment group could have had a previous treatment in any other hospital in the 2-year pre-time period. Thus, the initial treatment group predominantly, but not exclusively comprises incident cases of illness. Our reasoning behind this differentiation between these two groups was that potential intervention effects would have a different impact for IG/CG-patients who already had a treatment history at a FIT-/control hospital compared to IG/CG patients whose initial treatment took place after the onset of the FIT intervention. It gains even more importance considering that some of the FIT hospitals already have had specific contracts that to a certain extent exhibited FIT-like structures prior to initiation of the FIT programs. These already pre-existing contracts are likely to have facilitated the transition into the new FIT environment and could have already forestalled some of the intended intervention effects before FIT initiation [7, 12]. Hence, we expected more unbiased intervention effects to occur in the sub-cohort of the initial treatment group.

### Matching procedure

To minimize the likelihood of selection bias on the provider and patient level, we applied a two level matching algorithm. In a first step, to each FIT hospital, we allocated up to ten control hospitals in ranking order based on a priori defined knock-out criteria (i.e., same region, institutionalized structures, such as specialist departments, and PIA), criteria based on patients (i.e., number of cases per diagnosis) with a weighting of 50%, structural features of hospitals (e.g., number of beds or number of personnel) with a weighting of 25%, and regional factors (e.g., unemployment rate, household income) with a weighting of 25%. More details can be found in the already published study protocol [39]. In a second step, we applied a regression-based matching sequential two-fold algorithm on the level of patients for each FIT hospital. The procedure reduces the impact of possible confounding variables by leveling out IG and CG patient distributions regarding these exact variables. First, we matched patients exactly according to the variables year of study inclusion, initial or ongoing treatment, and type as well as number of mental disorders diagnosed at study inclusion. Thus, for these variables twin pairs of IG and CG patients had to exhibit the exact same value. Furthermore, propensity score matching was applied on variables sex, age at study inclusion and health care utilization before study inclusion (amount of inpatient care, day care, and outpatient utilization in PIA and established practitioners, all in the area of mental health care). The propensity matching procedure is based on a patient’s probability (i.e., propensity score) of group membership (IG/CG) which is calculated by logistic regression for the entire population. Patients’ propensity scores were utilized to determine twin pairs of IG and CG members based on a nearest neighbor algorithm (caliper = 0.25 standard deviation, without replacement). Hence, each patient of the IG was assigned its best fitting twin from the CG based on the least difference in value defined by the propensity score.

### Data and outcomes

We used claims data from over 70 different German SHIs covering >70 percent of all patients with mental disorders treated in FIT and control hospitals. In addition to sociodemographic characteristics (age and sex) and vital status, the data include comprehensive information on healthcare utilization in outpatient and inpatient sectors. The data include diagnoses (according to the International Statistical Classification of Diseases and Related Health Problems - German Modification, ICD-10-GM), procedures (according to the “Operationen-und Prozedurenschluessel,” OPS; German modification of the International Classification of Procedures in Medicine, ICPM), information on outpatient medical services (according to “Einheitlicher Bewertungsmassstab,” EBM), and prescribed medications (according to the German Anatomical Therapeutic Chemical (ATC) Classification). As all analyzed data were anonymous, the ethical committee of the University of Magdeburg confirmed that no ethical approval was necessary. Data were handled, analyzed, and reported according to Good Epidemiological Practice (GEP) [40], Good Practice of Secondary Data Analysis (GPS) [41], and the German Reporting Standard for Secondary Data Analyses, Version 2 (STROSA 2) [42]. Methodological, technical, and legal aspects of claims data analysis in EVA are described elsewhere [43].

In the analysis, we compared outcome differences between IG and CG of the patient-individual first year of the evaluation with respect to the patient-individual pre-time spanning over 1 year prior to study entrance. Outcomes were duration of inpatient care and sick leave. The first outcome describes the 1-year average cumulated length of hospitalization days. For the parameter sick leave, we aggregated the 1-year average cumulated number of days in sick leave, based on inpatient and outpatient sick leave prescriptions. Note, that there is an ambiguity on sick leave prescriptions including more than one diagnoses. We counted all sick leave days on prescriptions that included a patient’s index diagnosis. In addition, we counted every day a patient spent in inpatient care as sick leave day. The analysis was restricted to patients with “member” status as reported by the corresponding SHI.

### Analysis

We grouped patients into different clusters of mental disorders based on their primary ICD-10 diagnosis (index diagnosis, [44]) of the hospitalization case that constituted their inclusion into the study (see Table 1). We determined the index diagnosis using the diagnosis at hospital discharge. The grouping was done based on the most common disorder groups treated in psychiatric wards. Further, we also tried to balance out case numbers, avoiding clusters getting too small for inference. We excluded patients with more than one index diagnosis from the analysis to avoid confounding of effects due to non-exclusive diagnostic clusters. We analyzed the data using a three-level generalized linear mixed effect model utilizing a Quasi-Poisson distribution if the assumption of normality was violated. On Level one (level of measurements) the model contained a time factor variable (pre vs. 1st year after inclusion into the study). Level two (level of patients) comprised a group factor (IG vs. CG) as well as several patient-related covariates. These included our a priori-defined clusters of psychiatric disorders at study entrance, a measure of present mental comorbidities (defined as secondary F-diagnoses at discharge), as well as age and sex. Level three (hospital level) retained information about which one of the 18 FIT hospital (or its controls) the patient was assigned to. Primary measure of interest was the fixed interaction effect of *group* × *time*, adjusted for all covariates mentioned above. Since we wanted to estimate this effect for each of the diagnostic clusters separately, we estimated the three-way cross level interaction of *group* × *time* × *diagnostic cluster* for each of the outcomes. We used customized linear contrasts defining the average effect over all diagnostic clusters as reference category. Hence, we defined the diagnostic cluster-specific effect of the intervention as the arithmetical difference from the average effect over all diagnostic clusters (marginal effect). To further explore how the diagnostic clusters are related to psychiatric multi-morbidity we calculated the contingency coefficient C between diagnostic cluster and the comorbidity marker.

**Table 1:** A priori defined diagnostic clusters.

ICD-10	Diagnosis	Adult psychiatry	Child and adolescent psychiatry
F00–F03	Dementia	•	x
F10	Mental and behavioral disorders due to the use of alcohol	•	x
F20–F29	Schizophrenia, schizotypal and delusional disorders	•	x
F30–F39	Mood (affective) disorders	•	•
F43	Reaction to severe stress, and adjustment disorders	•	•
F40–F48	Neurotic, stress-related, and somatoform disorders	•	x
F60.31	Specific personality disorders of type borderline	•	x
F90–F98	Behavioral and emotional disorders with onset occurring in childhood and adolescence	x	•
All remaining F-codes	All other disorders	•	•

## Results

### Baseline characteristics

The overall cohort consisted of 36,571 individuals with 31,857 being adults (15,236 in initial treatment; 16,621 in ongoing treatment) and 4714 being children and adolescent patients (1754 in initial treatment; 2960 in ongoing treatment). In adults, more female patients were included while the opposite was the case in children and adolescents. Psychiatric comorbidity was low with roughly a quarter of patients having more than one psychiatric diagnoses (see Table 2). The rate of comorbidity was generally higher in the initial treatment sub-group compared to cases of ongoing treatment. In the later, the comorbidity rate was even lower in child and adolescent patients. However, within the sub-groups, mean age and sex, as well as comorbidity status was highly comparable between respective intervention and control samples due to the matching procedure.

**Table 2:** Baseline characteristics of all sub-cohorts.

	Adult psychiatry				Child and adolescent psychiatry			
	Initial treatment		ngoing treatment		Initial treatment		Ongoing treatment	
	Intervention (*N* = 7561)	Control (*N* = 7675)	Intervention (*N* = 8263)	Control (*N* = 8358)	Intervention (*N* = 872)	Control (*N* = 882)	Intervention (*N* = 1467)	Control (*N* = 1493)
Age								
Mean (SD)	50.3 (18.9)	50.6 (18.8)	52.1 (16.7)	52.8 (16.9)	11.7 (3.81)	11.8 (3.82)	11.4 (3.48)	11.8 (3.38)
Sex								
Male	3362 (44.5%)	3393 (44.2%)	3674 (44.5%)	3761 (45.0%)	498 (57.1%)	493 (55.9%)	957 (65.2%)	959 (64.2%)
Female	4199 (55.5%)	4282 (55.8%)	4589 (55.5%)	4597 (55.0%)	374 (42.9%)	389 (44.1%)	510 (34.8%)	534 (35.8%)
Index diagnosis								
Dementia	496 (6.6%)	509 (6.6%)	346 (4.2%)	371 (4.4%)	—	—	—	—
Mental and behavioral disorders due to use of alcohol	1357 (17.9%)	1373 (17.9%)	1062 (12.9%)	1096 (13.1%)	—	—	—	—
Mood (affective) disorders	2989 (39.5%)	3024 (39.4%)	3032 (36.7%)	3076 (36.8%)	107 (12.3%)	110 (12.5%)	99 (6.7%)	100 (6.7%)
Neurotic, stress-related, and somatoform disorders	527 (7.0%)	542 (7.1%)	421 (5.1%)	415 (5.0%)	—	—	—	—
Reaction to severe stress, and adjustment disorders	1112 (14.7%)	1128 (14.7%)	340 (4.1%)	343 (4.1%)	106 (12.2%)	109 (12.4%)	77 (5.2%)	78 (5.2%)
Schizophrenia, schizotypal and delusional disorders	707 (9.4%)	727 (9.5%)	2482 (30.0%)	2519 (30.1%)	—	—	—	—
Specific personality disorders of type borderline	89 (1.2%)	93 (1.2%)	190 (2.3%)	186 (2.2%)	—	—	—	—
Behavioral and emotional disorders in childhood and adolescence	—	—	—	—	566 (64.9%)	571 (64.7%)	1160 (79.1%)	1168 (78.2%)
Other disorders	284 (3.8%)	279 (3.6%)	390 (4.7%)	352 (4.2%)	93 (10.7%)	92 (10.4%)	131 (8.9%)	147 (9.8%)
No. of psychiatric comorbidities								
None	5827 (75.9%)	6005 (79.4%)	6182 (74.0%)	6161 (74.6%)	790 (89.6%)	725 (83.1%)	1144 (76.6%)	1088 (74.2%)
One	1322 (17.2%)	1174 (15.5%)	1605 (19.2%)	1569 (19.0%)	80 (9.1%)	132 (15.1%)	301 (20.2%)	332 (22.6%)
Two	387 (5.0%)	295 (3.9%)	430 (5.1%)	398 (4.8%)	11 (1.2%)	14 (1.6%)	41 (2.7%)	45 (3.1%)
More than two	139 (1.8%)	87 (1.2%)	141 (1.7%)	135 (1.6%)	1 (0.1%)	1 (0.1%)	7 (0.5%)	2 (0.1%)

Intervention = FIT hospital, Control = hospital employing routine care.

Initial treatment = patients that had no contact to the psychiatric ward or PIA in the corresponding FIT or control hospital in the 2 years prior to being included into the study.

Ongoing treatment = patients having at least one contact to the psychiatric ward or PIA in the corresponding FIT or control hospital in the 2 years prior to being included into the study.

### Main analysis

#### Adult psychiatry

**Fig. 1:**
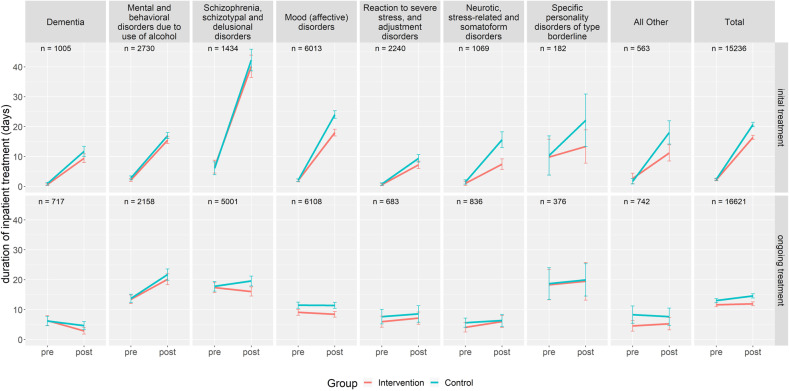
Mean duration of inpatient treatment in different clusters of psychiatric disorders (adult psychiatry). Intervention = FIT hospital, Control = hospital employing routine care; pre = 1 year prior to inclusion; post = 1 year after inclusion; Initial treatment = patients that had no contact to the psychiatric ward or PIA in the corresponding FIT or control hospital in the 2 years prior to being included into the study; Ongoing treatment = patients having at least one contact to the psychiatric ward or PIA in the corresponding FIT or control hospital in the 2 years prior to being included into the study.

In the initial treatment group, the average number of inpatient days sharply increased within the first year compared to pre-measurement in both IG and CG (see Fig. 1 and Table 3). Generally, both groups were homogeneous with regard to standard deviation. The average inpatient length of stay over both IG and CG was 18.6 days in the first year after inclusion into the study. It differed substantially between the diagnostic categories (see Fig. 1 and Table 4). The regression analysis revealed that especially patients with schizophrenia, schizotypal and delusional disorders (*b* = 17.9, *p* < 0.001) and patients with mood (affective) disorders (*b* = 3.6, *p* < 0.001) exhibited significantly more inpatient treatment utilization than the average of all clusters. All other clusters showed significantly fewer inpatient treatment days compared to average. Additionally, the time in inpatient treatment significantly increased with psychiatric multi-morbidity. Overall, there was a significant treatment effect of FIT programs compared to routine care, resulting in a smaller increase of 4.1 inpatient days (*b* = −4.1, *p* < 0.001). This effect was moderated by differences within the diagnostic clusters. While patients with mood (affective) disorders (*b* = −1.8, *p* < 0.05) and patients with neurotic, stress-related and somatoform disorders (*b* = −3.6, *p* < 0.05) showed an even stronger effect of the reduction of inpatient lengths of stay, the effect was significantly weaker in patients with mental and behavioral disorders due to use of alcohol (*b* = 3.3, *p* < 0.01). The correlation analysis revealed that mental and behavioral disorders due to use of alcohol were also positively associated with psychiatric multi-morbidity (*r* = 0.35, *p* < 0.001).

**Table 3:** Mean duration of inpatient treatment and mean duration of sick leave (adult patients only) in all sub-cohorts.

	Mean inpatient length of stay (SD)								Mean duration of sick leave (SD)							
	Initial treatment				Ongoing treatment				Initial treatment				Ongoing treatment			
	Intervention		Control		Intervention		Control		Intervention		Control		Intervention		Control	
	Pre	Post	Pre	Post	Pre	Post	Pre	Post	Pre	Post	Pre	Post	Pre	Post	Pre	Post
Adult psychiatry																
Dementia	0.7 (4.1)	9.4 (16.5)	1.0 (5.4)	11.7 (19.3)	6.3 (15.5)	2.9 (10)	6.2 (15.5)	4.7 (13.4)	1.2 (6.6)	9.0 (25.5)	0 (0)	11.4 (24.1)	8.6 (17.5)	8.4 (33.6)	6.4 (11.5)	6.9 (17.6)
Mental and behavioral disorders due to use of alcohol	2.2 (9.8)	15.5 (20.6)	2.9 (12.3)	17.0 (18.9)	13.5 (23.5)	20.2 (29.8)	13.8 (24.0)	21.7 (31.6)	12.6 (40.7)	43.9 (71.0)	13.9 (41.2)	45.6 (72)	28.3 (49.6)	38.5 (57.1)	27.2 (48)	41.7 (61.0)
Schizophrenia, schizotypal and delusional disorders	6.6 (29.1)	40.1 (50.9)	6.1 (29.4)	42.3 (49.6)	17.4 (41.8)	16.1 (38.8)	17.8 (41.1)	19.6 (42.2)	16.0 (50.8)	76.8 (89.8)	15.6 (49.3)	72.6 (84.3)	29.4 (61.9)	26.1 (55.8)	26.5 (55.9)	26.6 (58.1)
Mood (affective) disorders	2.0 (13.5)	18.0 (31.7)	2.1 (12.5)	24.0 (35.6)	9.1 (27.9)	8.4 (26.5)	11.5 (29.7)	11.4 (28.3)	32.3 (66.9)	98.9 (112.9)	33.1 (66.3)	98.3 (111.5)	52.2 (89.2)	46.9 (85.6)	47.6 (83.8)	46.3 (80.4)
Reaction to severe stress, and adjustment disorders	0.6 (5.9)	7.2 (21.0)	0.8 (6.8)	9.4 (21.6)	6.0 (17.6)	7.2 (19.4)	7.7 (23.1)	8.6 (26.0)	13.2 (40.3)	46.3 (82.6)	15.2 (46.9)	46.7 (84)	37.1 (69.2)	39.2 (71)	32.5 (60.9)	48.5 (97.7)
Neurotic, stress-related and somatoform disorders	1.1 (8.4)	7.5 (20.8)	1.5 (8.7)	15.6 (31.5)	4.1 (16.0)	6.0 (21.5)	5.6 (16.0)	6.4 (21.3)	29.4 (70.6)	73.6 (102.8)	20.5 (50)	69.6 (100.2)	33.6 (68.3)	27.6 (66.1)	27.9 (63.5)	39.1 (81.3)
Specific personality disorders of type borderline	9.8 (28.8)	13.4 (26.7)	10.4 (32.4)	22.1 (43.3)	18.3 (35.7)	19.5 (44.2)	18.7 (37.1)	20.0 (37.6)	21.0 (58.8)	39.4 (59.7)	25.5 (63.9)	41.3 (71.4)	37.0 (66.9)	43.5 (76.8)	32.4 (53.7)	33.4 (54.6)
All other	2.7 (15.1)	11.2 (23.2)	1.7 (7.5)	18.1 (33.2)	4.6 (17.8)	5.3 (20.7)	8.3 (27.6)	7.7 (27.7)	17.4 (54.3)	36.7 (80.6)	20.2 (51.8)	46.9 (80.9)	14.0 (45)	19.8 (57.9)	13.2 (38.9)	12.3 (41.9)
Total	2.2 (14.2)	16.4 (30.5)	2.4 (14.2)	20.7 (33.2)	11.6 (31.4)	11.9 (31.0)	13.0 (32.1)	14.6 (33.4)	22.5 (57.2)	71.0 (99.1)	22.9 (56)	70.9 (97.8)	37.0 (71.9)	36.1 (70.3)	33.7 (66.4)	37.3 (70.8)
Children and adolescent psychiatry																
Mood (affective) disorders	5.2 (26.9)	33.6 (50.7)	0.8 (8.5)	22.9 (40.4)	12.2 (32.9)	23.3 (46.5)	20.1 (40.8)	22.7 (43.9)								
Reaction to severe stress, and adjustment disorders	0.5 (3.8)	12.6 (26.9)	1.1 (11.2)	13.6 (34.9)	5.7 (17.3)	6.6 (21.3)	1.0 (6.2)	5.9 (23.8)								
Behavioral and emotional disorders in childhood and adolescence	0.9 (7.2)	8.2 (26.0)	0.9 (8.9)	11.3 (27.9)	3.0 (14.4)	4.1 (18.7)	4.0 (18.3)	6.3 (23.1)								
All other	0.9 (3.5)	15.8 (33.2)	3.2 (21.7)	30.7 (57.8)	11.6 (38.1)	10.4 (33.0)	5.9 (24.2)	12.7 (37.2)								
Total	1.4 (11.3)	12.7 (32.0)	1.2 (11.2)	15.1 (35.3)	4.5 (19.8)	6.1 (23.8)	5.1 (21.2)	8.0 (27.0)								

Intervention = FIT hospital, Control = hospital employing routine care.

pre = 1 year prior to inclusion; post = 1 year after inclusion.

Initial treatment = patients that had no contact to the psychiatric ward or PIA in the corresponding FIT or control hospital in the 2 years prior to being included into the study.

Ongoing treatment = patients having at least one contact to the psychiatric ward or PIA in the corresponding FIT or control hospital in the 2 years prior to being included into the study.

In the ongoing treatment group, patients generally had a different utilization behavior compared to the initial treatment group with already relatively high inpatient treatment in the pre-period (Fig. 1 and Table 4 in supplement). In the first year, the average time spent in hospital was 13.3 days, which is considerably lower than in the initial treatment group. There was also a significant interaction between the diagnostic categories. Patients with mental and behavioral disorders due to use of alcohol exhibited significantly more days in inpatient treatment than the average over all clusters (*b* = 6.4, *p* < 0.001). The inpatient length of stay was significantly longer among patients with psychiatric multi-morbidity. Patients in the IG also showed a significant treatment effect with fewer inpatient days compared to patients from the CG (*b* = −1.3, *p* < 0.01). There was no significant treatment effect between diagnostic clusters, though.

**Table 4:** Regression coefficients and their significance for selected variables taken from the regression model.

	Adult psychiatry								Child and adolescent psychiatry			
	Initial treatment				Ongoing treatment				Initial treatment		Ongoing treatment	
	Inpatient length of stay		Duration of sick leave		Inpatient length of stay		Duration of sick leave		Inpatient length of stay		Inpatient length of stay	
Intercept	1.2		−0.5		19.9	***	40.2	***	−9.0	***	−4.1	.
Group (control vs. intervention)	−0.2		−0.7		−1.3	**	3.7	*	0.3		−0.2	
Time (pre-time vs. post-time period)	18.3	***	48.0	***	1.6	***	3.7	***	13.9	***	2.9	***
Sex	0.4		5.4	***	0.3		3.8	**	2.7	**	2.7	***
Age	0.0	*	0.5	***	−0.1	***	−0.1	**	0.9	***	1.0	***
Psychiatric comorbidity	2.6	***	−12.8	***	8.2	***	−0.7		0.8		0.3	
Dementia	−7.5	***	−36.6	*	−3.1	*	−4.2		—	—	—	—
Mental and behavioral disorders due to the use of alcohol	−4.2	***	−16.4	***	6.4	***	−5.6	**	—	—	—	—
Schizophrenia, schizotypal and delusional disorders	17.9	***	9.0	*	0.2		−0.2		—	—	—	—
Mood (affective) disorders	3.6	***	17.2	***	−1.6	**	5.2	***	8.1	**	−0.4	
Reaction to severe stress, and adjustment disorders	−9.7	***	−16.6	***	−0.7		−5.7		−1.4		2.0	
Neurotic, stress-related, and somatoform disorders	−4.2	***	1.1		−0.8		−4.6	.	—	—	—	—
Specific personality disorders of type borderline	−6.5	*	−32.3	***	−0.3		−0.1		—	—	—	—
Behavioral and emotional disorders in childhood and adolescence	—	—	—	—	—	—	—	—	−3.5	*	−0.6	
All other diagnoses	−2.0		−21.4	***	−2.3		−3.1		13.6	***	4.0	.
Group × time	−4.1	***	0.4		−1.3	**	−4.6	**	−2.6	.	−1.3	
Group × time × dementia	2.1		−4.1		−0.6		3.8		—	—	—	—
Group × time × mental and behavioral disorders due to use of alcohol	3.3	**	−0.8		0.0		0.3		—	—	—	—
Group × time × schizophrenia, schizotypal and delusional disorders	1.4		3.3		−1.8	.	1.1		—	—	—	—
Group × time × mood (affective) disorders	−1.8	*	1.0		0.7		0.5		8.9	*	9.9	**
Group × time × reaction to severe stress, and adjustment disorders	2.1	.	1.2		1.5		−9.4		2.1		−0.6	
Group × time × neurotic, stress-related and somatoform disorders	−3.6	*	−5.3		2.5		−12.6	*	—	—	—	—
Group × time × specific personality disorders of type borderline	−4.1		2.2		1.2		10.0		—	—	—	—
Group × time × behavioral and emotional disorders in childhood and adolescence	—	—	—	—	—	—	—	—	−0.5		0.2	
Group × time × all other diagnoses	−3.7		−7.8		2.7		11.3	.	−10.0	*	−6.8	*

Significance codes: ***p < 0.001, **p < 0.01, *p < 0.05, ^.^*p* < 0.1.

Intervention = FIT hospital, Control = hospital employing routine care.

pre-time = one prior to inclusion; post-time = 1 year after inclusion.

Initial treatment = patients that had no contact to the psychiatric ward or PIA in the corresponding FIT or control hospital in the 2 years prior to being included into the study.

Ongoing treatment = patients having at least one contact to the psychiatric ward or PIA in the corresponding FIT or control hospital in the 2 years prior to being included into the study.

**Fig. 2:**
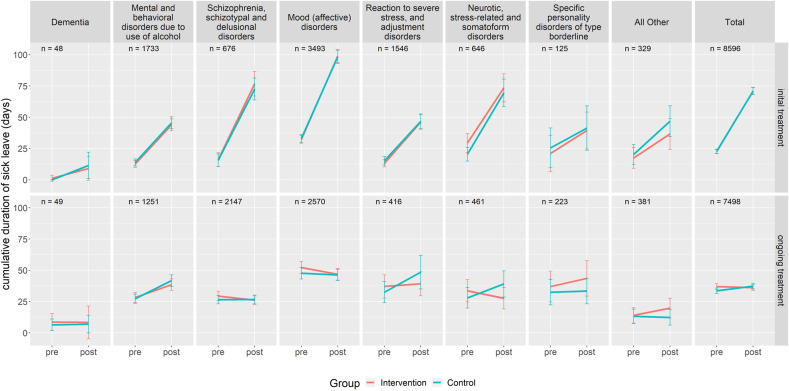
Mean duration of sick leave in different clusters of psychiatric disorders (adult psychiatry). Intervention = FIT hospital, Control = hospital employing routine care; pre = 1 year prior to inclusion; post = 1 year after inclusion; Initial treatment = patients that had no contact to the psychiatric ward or PIA in the corresponding FIT or control hospital in the 2 years prior to being included into the study; Ongoing treatment = patients having at least one contact to the psychiatric ward or PIA in the corresponding FIT or control hospital in the 2 years prior to being included into the study.

With respect to sick leave, there was a steep increase of days in sick leave between the pre-time and the post-time period in patients of the initial treatment group (see Fig. 2 and Table 3). Averaged over both IG and CG, and over all diagnostic clusters, the total number of days in sick leave was 70.9 days. Mean standard deviation was considerably higher compared to length of impatient stay but remained similar between both groups. Time spent in sick leave was significantly higher for patients with mood (affective) disorders (*b* = 17.2, *p* < 0.001 days) and patients with schizophrenia, schizotypal and delusional disorders (*b* = 9.0, *p* < 0.05). It was significantly lower than average in almost any other diagnostic cluster, especially in patients with dementia and patients with specific personality disorders of type borderline (see Table 4). Sick leave duration was also significantly lower in multi-morbid psychiatric patients (*b* = −12.8, *p* < 0.001). There was no significant treatment effect between intervention and control group.

In the ongoing treatment group the average number of sick leave days remained relatively stable from pre-time period to post-time period in both intervention and control group with 36.7 days in the first year after inclusion into the study (see Fig. 2 and Table 3). Sick leave was significantly higher in patients with mental and behavioral disorders due to use of alcohol and patients with adjustment disorders. Global treatment budgets reduced the duration of sick leave significantly by 4.6 days (*b* = −4.6, *p* < 0.01, see Table 4). The treatment effect was even higher in patients with neurotic, stress-related and somatoform disorders (*b* = −12.6, *p* < 0.05).

#### Child and adolescent psychiatry

**Fig. 3:**
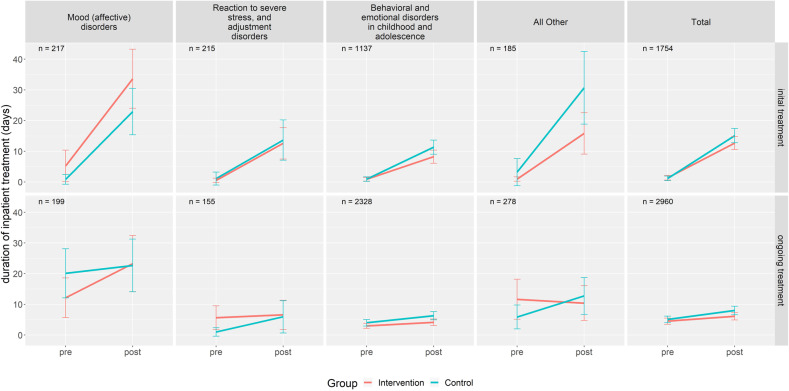
Mean duration of inpatient treatment in different clusters of psychiatric disorders (children and adolescent psychiatry). Intervention = FIT hospital, Control = hospital employing routine care; pre = 1 year prior to inclusion; post = 1 year after inclusion; Initial treatment = patients that had no contact to the psychiatric ward or PIA in the corresponding FIT or control hospital in the 2 years prior to being included into the study; Ongoing treatment = patients having at least one contact to the psychiatric ward or PIA in the corresponding FIT or control hospital in the 2 years prior to being included into the study.

In the initial treatment group, as already seen in adult patients, the average number of inpatient days increased within the first year post-time compared to the pre-time in both IG and CG (see Fig. 3 and Table 3). In the first year, the average inpatient length of stay was 13.9 days. Mean standard deviation between IG and CG was similar. Inpatient utilization in patients with mood (affective) disorders was significantly higher than average (*b* = 8.1, *p* < 0.05, see Table 4). Although there was no significant treatment effect of global treatment budgets when compared to routine care per se, inpatient treatment utilization was significantly higher in the intervention group compared to the control group in patients suffering from mood affective disorders (*b* = 8.9, *p* < 0.05).

In the ongoing treatment group, the average number of days in inpatient treatment in the first year was 7.1 days. There was no significant effect in hospitalization length in any diagnostic cluster. Further, there was no significant treatment effect between IG and CG. However, in the post-time period, the inpatient length of stay was considerably higher in the intervention group compared to the control group in patients in suffering from mood affective disorders (*b* = 9.9, *p* < 0.01).

## Discussion

### Utilization of inpatient hospitalization and sick leave in an initial treatment phase

There was an overall sharp increase in the initial treatment group from pre-period to post-period in both adult as well as child and adolescent patients regarding length of inpatient hospitalization. This increase most likely reflects the need for treatment of predominantly incident patients with mental disorders resulting in a high initial demand of care [45, 46].

In the initial treatment group, patients (not having contact to the index hospital in the previous 2 years) would not be affected by precursor contracts potentially forestalling some of the effects of FIT programs. Hence, we expected more unbiased intervention effects to occur in this sub-cohort. The duration of inpatient treatment was reduced by 4 days in adult patients in FIT hospitals compared to hospitals from routine care. This in itself is an important improvement as prolonged inpatient treatment might harm patients as they withdraw them from their normal living and social environment. Further, from previous analyses of a smaller sample of FIT hospitals we know that patients in FIT hospitals don't receive just less treatment. Rather, treatment is shifted from the inpatient sector towards day care or PIA treatment [33, 47]. The treatment effect was moderated by diagnostic cluster and was especially pronounced in adult patients with mood affective disorders as well as adult patients with neurotic, stress-related, and somatoform disorders. It was significantly lower than average (over all groups) in adult patients with mental and behavioral disorders due to the use of alcohol. This data shows a more prolonged inpatient treatment in FIT hospitals compared to routine care, which contradicts one of the key goals of FIT programs. Patients of IG and CG were matched according to their index diagnosis. This excludes alternative explanations such as having more cases of acute intoxications (F10) in the IG. However, given the coarse nature of claims data the nature of this effect remains elusive. On a more descriptive level it can be stated that the effect of a reduced length of inpatient hospitalization was generally low in patients with dementia, or mental and behavioral disorders due to use of alcohol, as well as patients with schizophrenia, schizotypal and delusional disorders due to use of alcohol. One might argue that these three clusters of mental diseases could be characterized to be specifically reliant on inpatient treatment procedures, leaving less room for reduction of inpatient hospitalization intervals. On the other hand, the strong intervention effect in patients with mood affective disorders as well as patients with neurotic, stress-related, and somatoform disorders could point to the fact that for these patient groups it is easier to reduce or fully avoid inpatient treatment. Alternatively, outpatient alternatives offered by FIT hospitals might have been more adequate for mood or neurotic, stress-related and somatoform disorders than for schizophrenia or dementia patients, and therefore FIT hospitals were more successful in reducing the length of hospitalization in these groups. This option will have to be explored in future analyses incorporating outpatient data. There was no present treatment effect of global treatment budgets with regard to the length of inpatient treatment in child and adolescent patients.

For adult patients, the observation of a steep increase from pre-time to the end of the first year also carries over to the duration in sick leave, which is highly plausible as inpatient treatment usually is associated with sick leave in the working population. There was no significant difference in sick leave duration between FIT hospitals and when contrasted to control hospitals from routine care.

### Utilization of inpatient hospitalization and sick leave in ongoing treatment

The overall average length of inpatient hospitalization in the first year was 13 days in adult patients and 7 days in child and adolescent patients. We found a significant reduction of inpatient hospitalization length in adults, however, with 1.3 days it was considerately smaller than the treatment effect in the initial treatment group. This phenomenon has already been explored in one of our previous works [31]. Many of the FIT hospitals had preexisting hospital structures that already implemented various flexible and integrated treatment approaches. Thus, some of the expected outcomes of FIT projects could have been forestalled and would be specifically present in patients with ongoing treatment cases. In child and adolescent patients, we did not detect a significant reduction in the length of inpatient hospitalization in FIT hospitals compared to controls.

Regarding sick leave duration, the cumulative average duration of sick leave at the end of the first year was about 37 days. We also identified a significant reduction in sick leave of 4.3 days in patients from FIT hospitals compared to patients from control hospitals. This effect seemed to be almost entirely driven by the sub-group of patients with neurotic, stress-related, and somatoform disorders. This is the first time, a significant reduction in the duration of sick leave as a mode of action of FIT programs is demonstrated. Together with the reduction in inpatient lengths of stay this result is of great importance, too, as it can provide indirect evidence that shorter hospitalization times do not harm patients but benefit their re-integration into everyday life.

## Strength and limitations

The study presented gathers data from FIT hospitals from locations all over Germany in total representing more than 300,000 patients [43]. The scientific use of claims data from SHI funds for the evaluation of new health care concepts has been established during the last years including analysis and reporting standards [41, 42]. Claims data offer complete and unbiased information on health care utilization [38]. However, validity of information on diagnoses in claims data can be a potential issue, especially regarding outpatient data [48]. Also, regarding sick leave prescriptions there remains some uncertainty about the working population within our data set. There is no information on working capacity of individuals per se. We decided to define the working population based on the insurance status of an individual being *member*, excluding pensioners. This operationalization tends to overestimate the number of people capable of working due to coding errors or because of a frequent long-term incapacity of work, independently of acute in- or outpatient treatment. However, please note that due to the control group design of the study we expect potential biases to affect each, IG and CG to equal amounts. While claims data offer essential information, they do not contain preference-based and patient-centered information such as symptom severity or functional level measures. Hence, it is important to stress that this study can only evaluate treatment success in FIT programs to a limited extend. In order to close this gap and gain such information the complementary evaluation projects PsychCare [49] and EVA_Tibas [33, 47] have been established. These projects will give access to patient-reported outcomes and patient-reported experience measures, such as changes in Quality of life, Satisfaction with care, symptom severity, or symptom recovery, by means of questionnaires and qualitative surveys. Additionally, claims data offer no deeper knowledge about working mechanisms of FIT implementations in individual hospitals. However, these kind of factors are not in the focus of our EVA64 study, as we are primarily interested in a global view by evaluating on outcomes that are expected to occur in every FIT hospital apart from their individual differences.

## Conclusion

We revealed that the implementation of a global treatment budget linked with the introduction of FIT programs is associated with shorter inpatient durations (both for adult patients in initial treatment and in ongoing treatment) as well as shorter durations in sick leave (only for adult patients in an ongoing treatment phase). The strength of the treatment effect concerning hospitalization varied across different diagnostic groups, leading to greater than average effects in patients with mood as well as neurotic, stress-related and somatoform disorders and lower effects in patients with dementia, schizophrenia, or abuse of alcohol. However, these two outcomes alone fall short of gaining a broad picture of the complex changes made by FIT programs. Although it is backed by other data that patients in FIT hospitals do receive alternative treatment (in the form of day care or PIA treatment) instead of less treatment, more research remains to be done. Future publications will also provide data on other outcomes like intensity of outpatient care, cross-sectoral treatment continuation, or inpatient re-admission rates.
